# Real-world use of thrombopoietic agents in treatment-naïve children with severe aplastic anemia: a multicenter retrospective study

**DOI:** 10.1038/s41598-026-51552-5

**Published:** 2026-05-11

**Authors:** Yasmine El Chazli, Gehad Abdallah, Marwa Zakaria, Ahmad Darwish, Gehan Khalifa, Tamer Hassan, Eslam Elhawary, Samar Elbahy, Mostafa Salama, Abdulhakim Alrawas, Yasser Wali, Maha Zeid

**Affiliations:** 1 Pediatric Hematology/Oncology Unit, Department of Pediatrics, Faculty of Medicine, Alexandria University, Alexandria, Egypt; 2 Pediatric Hematology and Oncology, Department of Pediatrics, Faculty of Medicine, Zagazig University, Zagazig, Egypt; 3 Pediatric Hematology/Oncology and Bone Marrow Transplantation Unit, Department of Pediatrics. Faculty of Medicine, Mansoura University, Mansoura, Egypt; 4 Pediatric Hematology and Oncology, Department of Pediatrics, Faculty of Medicine, Minia University, Minya, Egypt; 5 Pediatric Hematology, Oncology & BMT, Department of Pediatrics, Faculty of Medicine, Tanta University, Tanta, Egypt; 6 Pediatric Hematology, Pediatric Department, Faculty of Medicine, Benha University, Benha, Egypt; 7 Department of Child Health, College of Medicine and Health Sciences, Sultan Qaboos University, Muscat, Oman

## Abstract

Severe aplastic anemia (SAA) in children is a life-threatening disease. While hematopoietic stem cell transplantation (HSCT) remains the standard of care, many SAA patients lack a suitable donor. Thrombopoietin receptor agonists (TPO-RAs) have emerged as adjuncts to immunosuppressive therapy (IST). The objective of this study was to report a multicenter experience of TPO-RAs use in treatment-naïve children with SAA. We included 57 children, with a median age of 7 years (range 1.2–17), who received TPO-RA monotherapy (n = 13) or TPO-RA + cyclosporine A (CsA) (n = 44). Median hemoglobin, platelet counts, and absolute neutrophilic count all increased significantly in both groups (*p* < 0.001). Overall, 68.4% of patients achieved remission, and none reported severe adverse events related to the TPO-RA. The rate of remission was higher in the TPO-RA monotherapy group (92.3%) vs TPO-RA + CsA (59.1%), (*p* = 0.074), with a shorter median time to remission (6 vs. 12 months; *p* = 0.025). Median observation period was 24 months (range 4–108) with no significant difference between the two treatment groups. Treatment with TPO-RA monotherapy or TPO-RA + CsA resulted in clinically meaningful hematologic responses. These findings support prospective evaluation of TPO-RAs in frontline therapy for SAA when HSCT is not readily available, potentially changing management of pediatric SAA in specific contexts.

**Supplementary Information:**

The online version contains supplementary material available at 10.1038/s41598-026-51552-5.

## Introduction

Severe aplastic anemia (SAA) is a rare, life-threatening disorder characterized by pancytopenia and bone marrow (BM) hypocellularity. It disproportionately affects children and young adults and manifests significant clinical consequences such as bleeding, anemia, and infections^1,2^. Epidemiologically, the global incidence of SAA exhibits marked regional variations, with higher rates in Asia (5–6 cases/million/year) compared to Europe and North America (2–3 cases/million/year)^3–5^. The disease displays a triphasic age distribution, peaking in early childhood (2–5 years), young adulthood (20–25 years), and later adulthood (around 60 years)^6^.

Etiologically, SAA is categorized as either acquired or constitutional. The acquired form, more common and often immune-mediated, results from cytotoxic T-cell attacks on hematopoietic stem cells (HSCs), destroying them^7^. While most cases are idiopathic, some have been linked to environmental exposures; infections, particularly non-A, non-B, non-C hepatitis, can precede marrow aplasia in hepatitis-associated aplastic anemia (HAAA)^8^.

Historically, SAA was attributed to a polyclonal expansion of CD4 + T-cells and overproduction of inflammatory cytokines such as interferon-gamma (IFN-γ) and tumor necrosis factor-alpha (TNF-α)^9^. IFN-γ, produced by both immune and stromal cells, impairs HSCs function by inhibiting their growth and reducing self-renewal. This effect may occur through disruption of thrombopoietin and other growth factor signaling pathways, in part by acting as a decoy receptor. In addition, IFN-γ directly suppresses erythropoiesis by arresting HSCs at the early stages of differentiation^10^. Clinically, the manifestations of SAA in children are dictated by the degree of cytopenia, with symptoms such as mucocutaneous bleeding, infections, and fatigue frequently observed^7^.

Treatment strategies for children with SAA depend on patient age, donor availability, and disease severity. Hematopoietic stem cell transplantation (HSCT) from a fully matched sibling, related or unrelated donor remains the first-line therapy in pediatric patients, offering long-term survival rates exceeding 90% when performed early in the disease course^11^. In the absence of a matched donor, immunosuppressive therapy (IST) using anti-thymocyte globulin (ATG) combined with cyclosporine (CsA) is the standard approach and can lead to hematologic recovery in up to 70% of children. Supportive care, including transfusions and infection prophylaxis, plays a critical role during treatment^1^.

Recent advances have introduced the use of Thrombopoietin Receptor Agonists (TPO-RAs). Eltrombopag (EPG) showed promise in improving IST response rates in refractory or newly diagnosed patients without transplant options^12–15^. Some reports have also used romiplostim with promising results, and several clinical trials are currently underway in children and adults^16–20^. Further large-scale studies are needed to establish the long-term safety and efficacy of TPO-RAs, especially in the pediatric population, especially in resource-limited countries where access and availability of HSCT may be limited or delayed. This study aimed to assess efficacy of TPO-RAs in the management of treatment-naïve children with SAA, either monotherapy or in combination with CsA.

## Results

Seventy-nine files of patients with SAA were identified, after inclusion and exclusion criteria were revised, fifty-seven patients, diagnosed between 2016 and 2024, were included in this study from the different participating centers. Table 1 shows the demographic characteristics of patients. Viral serology was performed in all patients and was negative except in eight of them; EBV IgM was positive in one patient, and HAV IgM was positive in seven patients. Parvovirus serology was performed in only 20 patients and was negative in all. Flowcytometry for paroxysmal nocturnal hemoglobinuria (PNH) was performed in only 46 patients, and it was normal in all of them. The chromosomal breakage test was normal in all patients. None of the patients exhibited any clinical symptoms or signs suggestive of an inherited bone marrow failure syndrome or PNH.

**Table 1:** Demographic and initial clinical characteristics of the study population.

	All patients N = 57	TPO-RA only N = 13	TPO-RA + CsA N = 44	*p*-value
Country				
Oman	7 (12.3%)	7 (53.8%)	0 (0%)	< 0.001
Egypt	50 (87.7%)	6 (46.2%)	44 (100%)	
Sex				
Males	30 (52.6%)	6 (46.2%)	24 (54.5%)	0.754
Females	27 (47.4%)	7 (53.8%)	20 (45.5%)	
Clinical findings				
Pallor	54 (94.7%)	12 (92.3%)	42 (95.5%)	1.000
Bleeding manifestations				
None	6 (10.5%)	3 (23.1%)	3 (6.8%)	0.209
Mild	39 (68.4%)	8 (61.5%)	31 (70.5%)	
Moderate	10 (17.5%)	1 (7.7%)	9 (20.5%)	
Severe	2 (3.5%)	1 (7.7%)	1 (2.3%)	
Fever	29 (50.9%)	7 (53.8%)	22 (50.0%)	1.000
Sepsis	15 (26.3%)	1 (7.7%)	14 (31.8%)	0.168
Evidence of hepatitis	10 (17.5%)	0 (0%)	10 (22.7%)	0.139
Classification of aplastic anemia				
Severe	38 (66.7%)	7 (53.8%)	31 (70.5%)	0.435
Very severe	19 (33.3%)	6 (46.2%)	13 (29.5%)	
Age at diagnosis (years)				
Median (range)	7 (1.2–17)	7 (1.7–11)	8 (1.2–17)	0.145
BM cellularity (%)				
Median (range)	10 (5–30)	10 (10–22.5)	10 (10–15)	0.870

*N* number, *Min–Max* minimun-maximum, *SD* standard deviation, *BM* bone marrow.

**Fig. 1:**
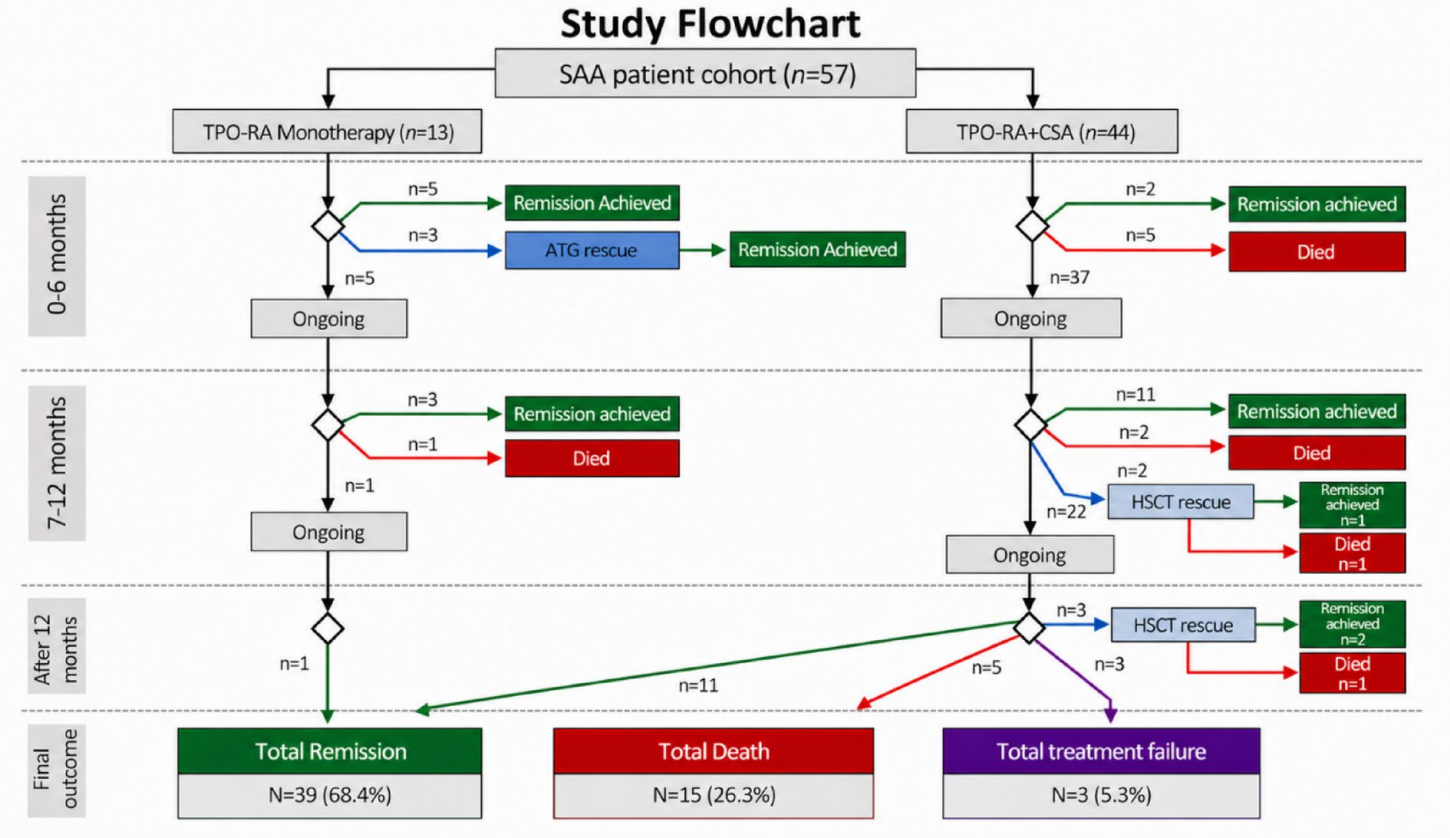
Study flowchart.* CsA* cyclosporine,* TPO-RA* thrombopoietin receptor agonist,* SAA* severe aplastic anemia,* HSCT* hematopoietic stem cell transplantation, *ATG* antithymocyte globulin, *n* number. The two patient groups are shown from the top, from the time of SAA diagnosis (and the start of treatment) through follow-up intervals until the final outcome. The bottom boxes show the total number and percent of patients who achieved remission or died (including those who did after rescue therapy), or who had treatment failure and continued on supportive care.

**Fig. 2:**
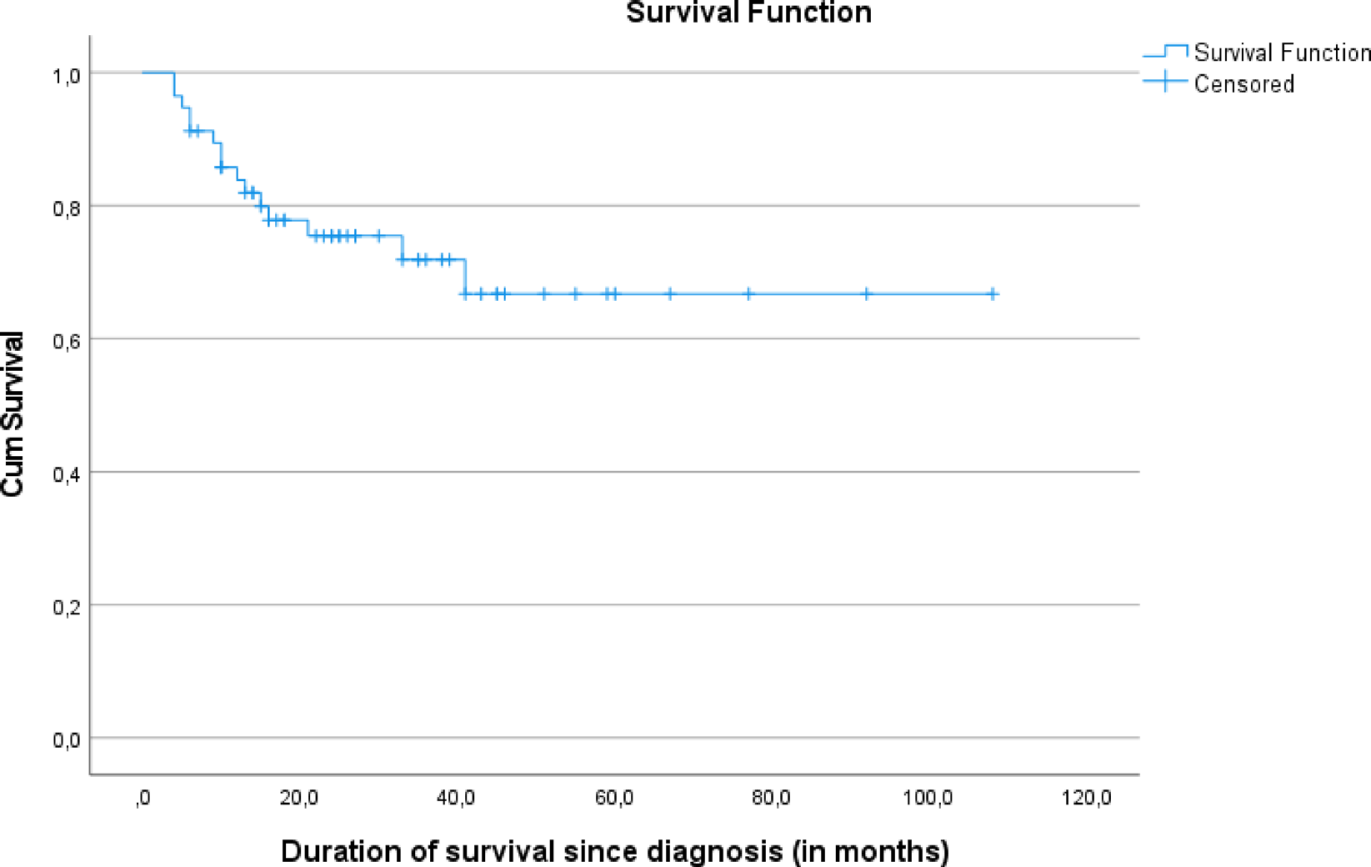
Kaplan Meier survival curve of the study population.

**Fig. 3:**
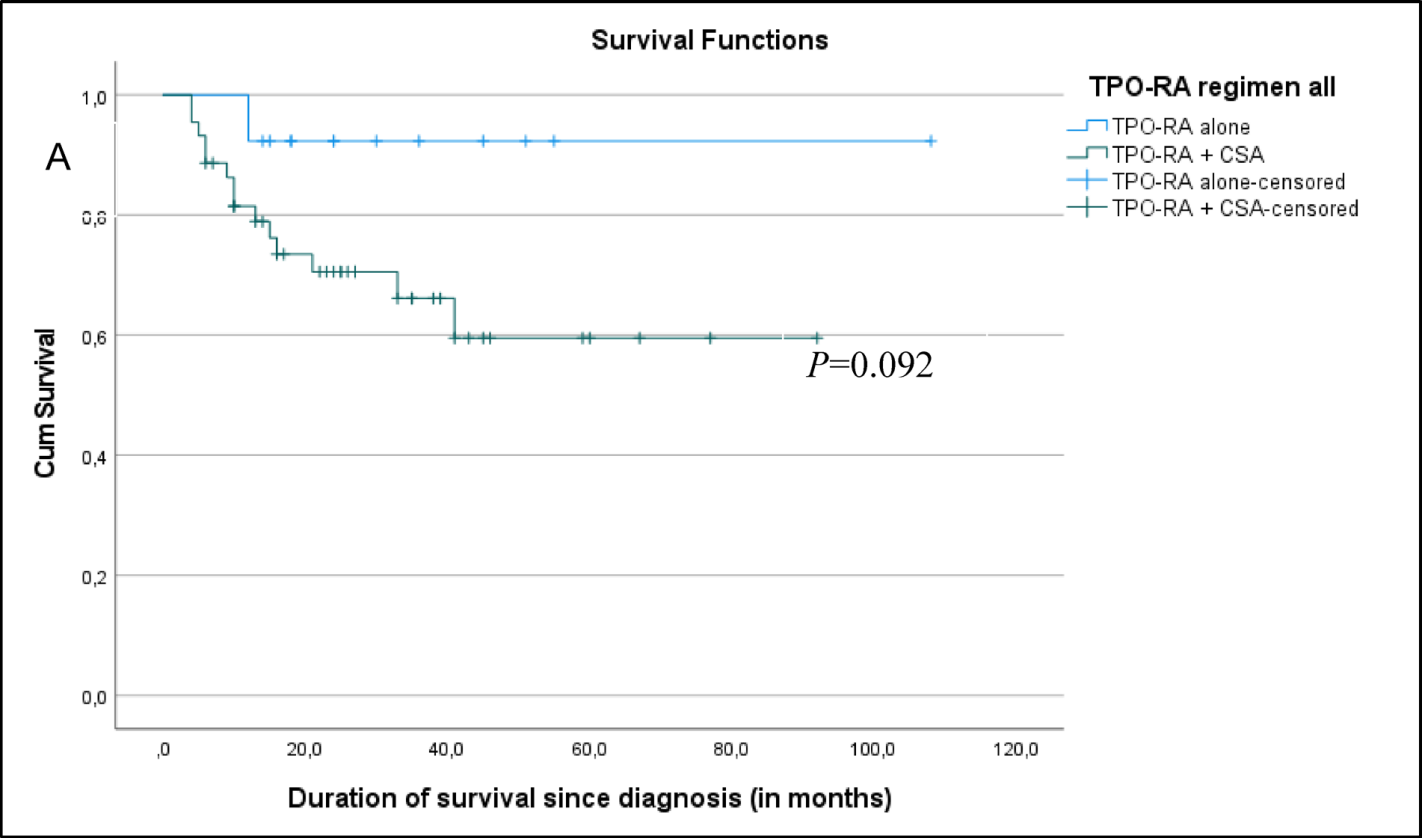

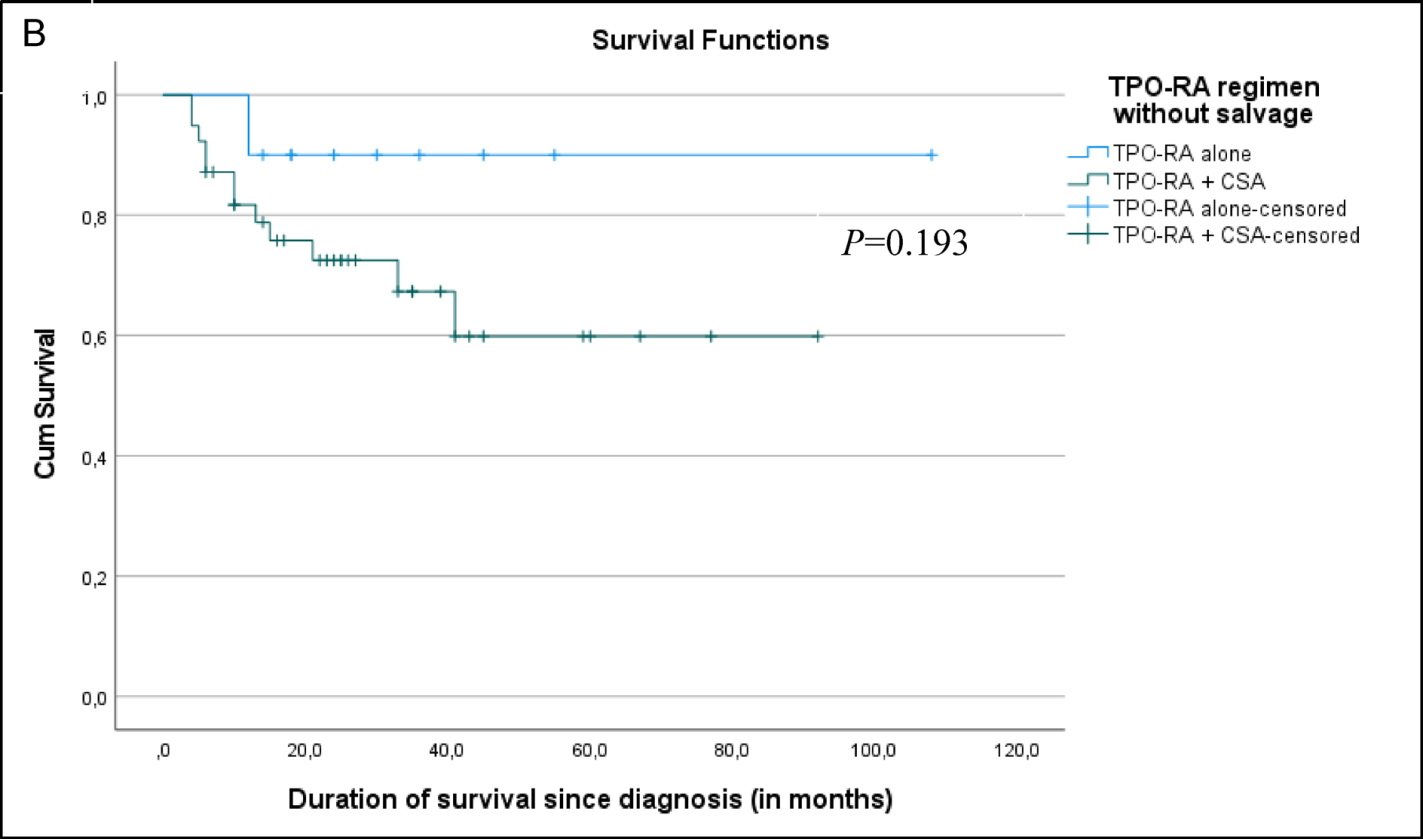
Kaplan Meier survival curve comparing TPO-RA alone vs TPO-RA+CsA. **A** Survival curve comparing the final outcome of TPO-RA monotherapy vs TPO-RA + Cs. **B** Survival curve comparing TPO-RA monotherapy vs TPO-RA + CsA groups’ outcome without salvage therapy.

The hematological parameters at baseline and at the last follow-up are shown in Table 2. As shown in the study flow chart (Fig. 1), patients were categorized according to the treatment they received into the TPO-RA monotherapy or the TPO-RA + CsA treatment group. All patients from Oman received Romiplostim as a TPO-RA monotherapy regimen, while patients from Egypt received Eltrombopag monotherapy or in combination with CsA, according to the local protocol of each centre and the availability of the drug. Table 3 presents the treatment and patient outcomes, and Figs. 2, 3A and B show the survival curves for patients and a comparison of survival between the TPO-RA monotherapy and TPO-RA + CsA groups. Twenty-four (42.1%) patients did not respond to the initial TPO-RA-based regimen after 12 weeks of treatment; when available, they received salvage therapy, in the form of horse ATG (3 patients, 12.5%) or HSCT (5 patients, 20.8%) from a fully matched sibling donor (2 patients) or haploidentical parent (3 patients). The delay of salvage therapy was essentially due to the time required to complete investigations to exclude an underlying inherited bone marrow failure syndrome and search for an adequate HSCT donor. A total of 15 deaths occurred in the present study; the 5-year overall survival was 74%, estimated using the Kaplan–Meier method. The most common cause of death was infection (9 patients, 60%), including one during HSCT; the most frequently reported organisms were multidrug-resistant Klebsiella and Enterobacter species, and in one patient, Burkholderia cepacia was cultured from the blood. The three patients, refractory to the TPO-RA regimen for whom no salvage therapy was available, have been alive and maintained on supportive care for 7, 23, and 27 months, respectively. Moreover, 10 patients received G-CSF as supportive therapy but showed no significant response. None of the patients receiving TPO-RA reported any severe adverse events (AE) related to the drug, and the most common reported AE were mild gastrointestinal discomfort and mildly elevated liver enzymes, but none of the patients had to interrupt the treatment due to these AE. Only five patients underwent follow-up bone marrow examination after TPO-RA was discontinued, and none showed increased reticulin. Table 4 shows the treatment and outcomes for the HAAA patient subgroup, and supplementary Table 1 compares EPG and romiplostim monotherapy.

**Table 2:** Hematologic parameters of the study population at baseline and at last follow-up.

	Baseline hematological parameters				Hematological parameters at last follow-up*				*p*-value^#^
	All patients N = 57	TPO-RA N = 13	TPO-RA + CsA N = 44	*p*-value	All patients N = 49	TPO-RA N = 10	TPO-RA + CsA N = 39	*p*-value	
Hb (g/L) Median (range)	72 (20–99)	73 (20–99)	68 (30–97)	0.829	110 (24–149)	115 (24–134)	106 (25–149)	0.256	< 0.001
Retics (%) Median (range)	0.3 (0.1–2)	0.11 (0.1–2)	0.3 (0.1–1)	0.513	1.35 (0.2– 3.75)	1.35 (0.6–2.1)	1.4 (0.2–3.75)	1.000	< 0.001
Plt (10^9^/L) Median (range)	11 (2–67)	13 (3–67)	11 (2–60)	0.943	105 (0–281)	155 (2–208)	70 (0–281)	0.256	< 0.001
TLC (10^9^/L) Median (range)	2.6 (0.6–7)	3 (1.4–5.5)	2.5 (0.6–7)	0.396	5 (1.1–10.18)	5.85 (1.5–10.2)	4.87 (1.1–10.1)	0.669	< 0.001
ANC (10^9^/L) Median (range)	0.30 (0–2.3)	0.28 (0–2.1)	0.30 (0 –2.3)	0.734	1.8 (0.0–5.8)	2.41 (0.4–4.1)	1.6 (0–5.8)	0.113	< 0.001
ALC (10^9^/L) Median (range)	1.84 (0.2–5.3)	1.6 (0.2–5.3)	1.97 (0.36–4.4)	0.575	2.57 (0.32–5.8)	2.35 (1–5.8)	2.75 (0.32–4.8)	0.719	**0.013**

*N* number, *Hb* hemoglobin, *Retics* reticulocytes, *Plt* platelets, *TLC* total leukocyte count, *ANC* absolute neutrophil count, *ALC* absolute lymphocyte count, *CsA* cyclosporine, *TPO-RA* thrombopoietin receptor agonist monotherapy.

*Patients who received salvage therapy were excluded ^#^The Wilcoxon Signed-Rank test was used to compare the baseline and follow-up hematological parameters of all patients.

**Table 3:** Outcomes and survival of the study population.

	All patients N = 57	TPO-RA only N = 13	TPO-RA + CsA N = 44	*p*-value
Outcome of TPO-RA-based regimen				
Remission	33 (57.9%)	9 (69.2%)	24 (54.5%)	0.567
Failure of treatment	11 (19.3%)	3 (23.1%)	8 (18.2%)	
Death	13 (22.8%)	1 (7.7%)	12 (27.3%)	
Remission at 6 months	7 (12.3%)	5 (38.5%)	2 (4.5%)	0.008
Remission at 12 months	22 (38.6%)	8 (61.5%)	14 (31.8%)	0.191
Salvage therapy	8 (14%)	3 (23.1%)	5 (11.4)	0.539
Final outcome				
Remission	39 (68.4%)	12 (92.3%)	27 (61.4%)	0.074
Failure of treatment	3 (5.3%)	0 (0%)	3 (6.8%)	
Death	15 (26.3%)	1 (7.7%)	14 (31.8%)	
Cause of death	N = 15	N = 1	N = 14	0.402
Infections	9 (60%)	0 (0%)	9 (64.3%)	
Bleeding	2 (13.3%)	0 (0%)	2 (14.3%)	
Unreported	3 (20%)	1 (100%)	2 (14.3%)	
HSCT failure	1 (6.7%)	0 (0%)	1 (7.1%)	
Duration till remission on TPO-RA-based regimen (months)	N = 33	N = 9	N = 24	**0.025**
Median (range)	10 (3–36)	6 (3–14)	12 (6–36)	
Observation period (months)				0.829
Median (range)	24 (4–108)	24 (12–108)	23.5 (4–92)	

TPO-RA, thrombopoietin receptor agonist; CsA, cyclosporine; HSCT: hematopoeitic stem cell transplantation.

**Table 4:** Comparison of patients’ characteristics, treatment and outcome between HAAA and idiopathic SAA patients.

	HAAA N = 10	Idiopathic SAA N = 47	*p*-value
Age at diagnosis (years)			
Median (range)	8.5 (3.5–17.0)	7 (1.2–15.5)	0.992
Duration till remission on TPO-RA-based regimen (months)			
Median (range)	N = 6 16 (7–35)	N = 27 10 (3–36)	0.173
Observation period (months)			
Median (range)	16.5 (7–67)	25 (4 –108)	0.456
Treatment received			
TPO-RA	0 (0%)	13 (28.7%)	0.096
TPO-RA + CsA	10 (100%)	34 (72.3%)	
Outcome of TPO-RA-based regimen			
Remission	6 (60%)	27 (57.4%)	0.802
Failure of treatment	3 (30%)	10 (21.3%)	
Death	1 (10%)	10 (21.3%)	
Received salvage therapy	2 (20.0%)	6 (12.8%)	0.619
Final outcome			
Remission	6 (60%)	33 (70.2%)	1.000
Failure of treatment	1 (10%)	2 (4.3%)	
Death	3 (30%)	12 (25.5%)	

*HAAA* hepatitis associated aplastic anemia, *SAA* severe aplastic anemia, *TPO-RA* thrombopoietin receptor agonist, *CsA* cyclosporine.

## Discussion

This multicenter retrospective study provides real-world evidence on the use of TPO-RAs as frontline therapy in treatment-naïve children with SAA, especially where access to HSCT and ATG remains limited. While TPO-RAs are well established in the treatment protocols for SAA in adults, pediatric evidence, particularly outside clinical trial settings, remains scarce. Our findings, therefore, make a meaningful contribution to the literature as we analyzed the outcomes of 57 children with SAA treated with TPO-RA–based regimens, either as monotherapy or in combination with CsA. At baseline, the two treatment groups were well-matched, showing no significant differences in any hematological parameter. Consistent with prior reports, the majority of patients (68.2%) achieved hematologic remission, and overall survival was favorable. EPG, when added to standard IST, has been shown to improve response rates and hematologic recovery in both adults and children with SAA^13^. Most of the earlier studies added TPO-RA to the standard IST regimen of ATG and CsA.^19,21^ In a recent single-center Egyptian study, CsA monotherapy was compared to EPG + CsA, and both groups showed favorable responses with better overall outcome in the combination group^22^. A recently published retrospective Turkish study also confirmed a faster response when adding EPG to IST compared to IST monotherapy, without increasing toxic effects^23^. All this data supports that the addition of EPG in pediatric SAA patients leads to better outcomes.

Importantly, we observed that children receiving TPO-RA monotherapy achieved remission significantly earlier than those receiving combined therapy (6 vs. 12 months, *p* = 0.025), with 38.5% of the TPO-RA monotherapy group achieving remission by 6 months vs 4.5% among the TPO-RA + CSA, (*p* = 0.008); long-term survival did not differ between the two groups. Although there is no clear explanation for this observation, we think it could prompt the design of randomized controlled trials comparing the efficacy and time to response between the two treatment regimens. In a recent publication from China, three groups of SAA patients were assigned to standard IST + EPG, EPG + CSA, or CSA monotherapy; although patients on standard IST + EPG achieved the highest overall response, those on EPG + CSA achieved remission (complete and partial) earlier than the two other groups (not statistically significant) ^24^. This may support the fact that the value of CSA as the only immunosuppressive drug in SAA is minimal. Moreover, patients who received EPG or Romiplostim monotherapy achieved high remission rates (83.3% and 57.1%, respectively) with excellent final outcomes. Our findings suggest that romiplostim monotherapy, like EPG, may accelerate hematologic recovery, supporting recent pediatric case series and adult trials demonstrating the promising efficacy of TPO-RA monotherapy ^17,25^. Larger prospective randomised trials are required to confirm these preliminary clinical observations.

Follow-up blood counts showed improvements in hemoglobin levels, platelet counts, and absolute neutrophilic count (ANC) in both treatment groups. Patients receiving TPO-RA monotherapy had higher platelet counts at the last follow-up compared to the TPO-RA + CsA group (median 155 vs. 70 × 10^9^/L), although this difference was not statistically significant (*p* = 0.256). Additionally, a higher percentage of patients achieved remission in the TPO-RA monotherapy group either before (69.2% vs. 54.5%) or after salvage therapy (92.3% vs. 61.4%). Fewer patients died in this group (7.7% vs. 31.8%), but these differences did not reach statistical significance (*p* = 0.567 and 0.074, respectively). It is important to note that earlier studies of children with SAA from Egypt showed relatively poor responses compared to international reports. In a retrospective study of 23 children treated with IST (some received ATG and CsA, others CsA monotherapy), the overall survival was 43%^26^. In another study including 60 children, those who received IST (either CsA monotherapy, CsA and EPG, or ATG + CsA + EPG) and patients who underwent HSCT had an overall response rate of 70%^27^.

The median observation period for all patients was 24 months. The estimated 5-year overall survival was 74%; although there was a strong trend toward improved survival in the TPO-RA monotherapy group compared to the TPO-RA + CsA group, both before and after salvage therapy, this did not reach statistical significance (*p* = 0.092 and 0.193, respectively). Despite encouraging response rates, one-quarter of patients died, with infection being the leading cause of mortality. This finding underscores the importance of supportive care, including infection prophylaxis and early intervention strategies, particularly during the period of profound cytopenia before hematologic recovery, including monitoring for reactivation of viral infections. The inferior outcome in SAA patients in low- and middle-income countries is partly due to delays in HSCT, which is limited by donor availability, infrastructure constraints, and financial barriers. Moreover, ATG is costly, inconsistently available, and requires intensive monitoring. In such contexts, treatment delays of more than three months are common. TPO-RA–based regimens provide a pragmatic alternative that may bridge the therapeutic gap for children awaiting definitive treatment or for those who cannot access standard IST.

In the present study, ten patients (17.5%) had HAAA. They all received TPO-RA + CsA with a remission rate of 60%. There was no significant difference in the mortality in children with HAAA compared to those with idiopathic SAA (30% vs. 25.5%, respectively). Previous reports suggest similar responses to IST and TPO-RAs in children with HAAA^28^.

Over the past few years, EPG added to the IST has been reported to have an acceptable safety profile and promising results^29^. Nevertheless, the latest published pediatric recommendations for the management of idiopathic SAA recommend IST (ATG + CsA) as the frontline in children without a matched sibling donor. They concluded that the data were insufficient for recommending upfront addition of TPO-RA (mainly EPG) in the pediatric population, while it is recommended in the adult population. For romiplostim, it stated that data are still emerging but are less established. In contrast, the addition of TPO-RA to IST is recommended for children with refractory SAA as a second-line treatment^30,31^. Real-world studies, like the present study, are crucial for reporting the safety and efficacy of TPO-RA in the management of pediatric SAA. The accumulation of case series of such a rare disease may help advance the treatment recommendations. Moreover, other emerging agents, like avatrombopag and hetrombopag, which show improved safety and more convenient dosing schedules, may further modify the approach to the Treatment of patients with SAA^32–35^. The favorable safety profile observed in this study further supports the feasibility of TPO-RA use in frontline settings. None of the patients treated with TPO-RA required discontinuation of treatment due to adverse events or experienced SAE related to the drug. These findings reinforce the growing body of evidence indicating that TPO-RAs are well-tolerated in pediatric populations^29^.

The present study has limitations, including its retrospective design, small sample size, and heterogeneity in treatment allocation, which may introduce selection bias. However, given the rarity of SAA, especially in pediatric populations, these real-world multicenter data offer valuable insight that complements controlled trial evidence. In conclusion, TPO-RA–based regimens appear as an effective therapeutic option in pediatric SAA, without the need to add CsA, especially in countries where access to HSCT and ATG may be limited or delayed. Although survival differences were not significant, the clinical trend toward lower mortality and faster hematologic recovery in the monotherapy group is noteworthy, especially in resource-limited settings where prolonged cytopenia significantly increases risks of sepsis, bleeding, and early mortality.

## Methods

The study was conducted in accordance with the principles of the Declaration of Helsinki. Ethics approvals were obtained from the Alexandria University Ethics Committee (IRB Number 00012098), Faculty of Medicine, Alexandria University, Egypt and the Medical Research and Ethics Committee, College of Medicine, Sultan Qaboos University, Muscat, Oman. Informed ascent/consent were obtained from all the patients/legal guardians before the study. Patient data were collected retrospectively from medical records, and confidentiality was maintained. The inclusion criteria were children aged 1–18 years at the time of diagnosis of SAA. Eligible patients had idiopathic SAA, diagnosed by a bone marrow biopsy showing cellularity < 25–30% in association with at least two clinically significant cytopenias: ANC < 0.5 × 10^9^/L/μL, platelet count < 20 × 10^9^/L or platelet-transfusion dependence, reticulocyte count < 60 × 10^9^/L, hemoglobin < 90 g/L, or red cell transfusion dependence. Aplastic anemia was considered very severe (VSAA) if the ANC was < 0.2 × 10^9^/L/μL. They had a negative chromosomal breakage test and PNH screen. All patients had normal renal and liver functions prior to treatment initiation. Patients were prescribed a TPO-RA-based medical treatment if HSCT was unavailable, or unsuitable as upfront therapy, or declined by caregivers, or if a delay of more than 3 months was expected before HSCT. The treatment was started within 4 weeks of the diagnosis. The doses of TPO-RAs and CsA were calculated based on the patient’s age and weight. CsA drug level was routinely monitored to ensure a therapeutic level was achieved and maintained (trough plasma concentration at 100–150 ng/mL). Any patient who did not fulfill these criteria was excluded from the present study. Patients with neutropenia were maintained on prophylactic trimethoprim/sulfamethoxazole or pentamidine. Voriconazole was added during periods of profound neutropenia. Patients were admitted and treated for febrile neutropenia according to the local hospital’s policy. Patients received irradiated packed red blood cell and/or platelet transfusions when clinically indicated. A complete hematologic response was defined as neutrophil > 1 × 10^9^/L, platelet > 100 × 10^9^/L, and hemoglobin > 100 g/L. For patients who did not respond to the TPO-RA-based regimen, salvage therapy options included ATG or HSCT.

Data were collected from multiple centers in Egypt (University Hospitals of Alexandria, Mansoura, Menia, Zagazig, Benha, and Tanta) and from Sultan Qaboos University Hospital in Muscat, Oman. Medical records of patients meeting the inclusion criteria were reviewed. Extracted data included demographic information, presenting symptoms and signs, hematologic parameters at diagnosis and last follow-up, bone marrow biopsy findings, and viral serologies (HIV, EBV, CMV, hepatitis A, B, and C, and parvovirus B19). Patients were followed up while on treatment, and the date of the last follow-up or the date of death was used to calculate the observation period and report outcomes. Data was fed to the computer and analyzed using IBM SPSS Statistics for Windows, version 20.0 (IBM Corp., Armonk, NY, USA). Continuous variables were summarized as mean ± standard deviation (SD) or median (range), depending on distribution assessed using the Shapiro–Wilk test. Categorical variables were described as counts and percentages. Group comparisons were conducted using the independent samples t-test for normally distributed data and the Mann–Whitney U and Wilcoxon Signed-Rank tests for non-normally distributed data. Associations between categorical variables were evaluated with the Chi-square test or Fisher’s exact test when expected cell counts were < 5. Survival was estimated using the Kaplan–Meier method, and differences between treatment groups were compared using the log-rank (Mantel–Cox) test. All p-values were two-sided, and values < 0.05 were considered statistically significant. During the preparation of this work, the authors used ChatGPT for language editing and to create the diagram in Fig. 1. After using this tool, the authors reviewed and edited the content as needed and take full responsibility for the content of the publication.

## Supplementary Information

Supplementary Information.

## Data Availability

Data is available on reasonable request.
