# Supplementary material for: Real-world use of thrombopoietic agents in treatment-naïve children with severe aplastic anemia: a multicenter retrospective study
Source: Sci Rep. 2026 May 11;16:14723. doi: 10.1038/s41598-026-51552-5 (PMC13161418; doi:10.1038/s41598-026-51552-5)
Supplement: Supplementary file 1 — Supplementary Information. [file 41598_2026_51552_MOESM1_ESM.docx]

|  | **EPG**  **N=6** | **Romiplostim**  **N=7** | ***p*-value** |
| --- | --- | --- | --- |
| **Age at diagnosis** (years)  Min-Max  Mean ± SD | 2.5-11.0  6.68±2.9 | 1.7-10  5.96±2.84 | 0.781 |
| **Duration till remission on TPO-RA-based regimen** (months)  - Median (range) | N=5  7 (3-14) | N=4  6 (5-8) | 0.524 |
| **Duration of survival since diagnosis** (months)  - Median (range) | 27 (12-45) | 24 (14-108) | 1.000 |
| **Outcome of TPO-RA-based regimen**  Failure of treatment  Remission  Death | 0 (0%)  5 (83.3%)  1 (16.7%) | 3 (42.9%)  4 (57.1%)  0 (0%) | 0.190 |
| **Received salvage therapy** | 0 (0%) | 3 (42.9%) | 0.243 |
| **Final outcome**  Failure of treatment  Remission  Death | 0 (0%)  5 (83.3%)  1 (16.7%) | 0 (0%)  7 (100%)  0 (0%) | 0.936 |

**TABLE 1 supp. Comparison of patients' characteristics, treatment and outcome between EPG and Romiplostim monotherapy in SAA patients**

N: number; EPG: eltrombopag; Min-Max: minimun-maximum; SD: standard deviation; CsA: cyclosporine; TPO-RA: thrombopoietin receptor agonist monotherapy.
